# Impact of the COVID-19 Stay-at-Home Order on Diet and Health in Community-Dwelling Older Adults With Obesity

**DOI:** 10.1093/geroni/igab046.1775

**Published:** 2021-12-17

**Authors:** Kathryn Porter Starr, Michael Borack, Marshall Miller, Heather Hutchins-Wiese, Alyssa King, Kenlyn Young, Connie Bales

**Affiliations:** 1 Duke University School of Medicine, Hillsborough, North Carolina, United States; 2 Duke University School of Medicine, Durham, North Carolina, United States; 3 Eastern Michigan University, Ypsilanti, Michigan, United States

## Abstract

Profound restrictions were placed on previously free-living older adults due to mandatory stay-at-home orders for Covid-19. Recognizing the potential for worsening health and heightened risk of Covid-19 complications with older age and obesity, we conducted a survey to assess the impact of stay-at-home requirements on diet, health/social behaviors, and food security in 58 older adults (age=70.8±6.2, 55% Black, 93% female) who had participated in past obesity-reduction trials. A 71-item questionnaire was administered by phone and included demographics, health, lifestyle and dietary habits, food attitudes, and food security questions. Results showed indicators of heightened health risk, including health care appointments either delayed/cancelled (69%) and self-reported weight gain (62%). Of those with weight gain, 22% reported a gain of >10 pounds (33% gained 5-10 pounds and 7% < 5 pounds). Increased food intake was reported by 67% and 45% felt their eating patterns were less healthy due to increased snacking (71%) and consumption of sweets (41%). Food access and isolation were also an issue, as 51% were concerned about leaving the house for food and 81% reported eating alone. While some positive behaviors were reported (new ways to access food and health information, more reported cooking at home), the majority of findings indicated increased risk of obesity and its complications. The findings emphasize the need for effective lifestyle interventions that can be delivered remotely to high-risk older adults; this would benefit those presently self-restricted for Covid-19 concerns as well as other isolated older adults who need better access to individualized interventions.

